# The Role of Hydrocolloids in Gluten-Free Bread and Pasta; Rheology, Characteristics, Staling and Glycemic Index

**DOI:** 10.3390/foods10123121

**Published:** 2021-12-16

**Authors:** Alina Culetu, Denisa Eglantina Duta, Maria Papageorgiou, Theodoros Varzakas

**Affiliations:** 1 National Institute of Research & Development for Food Bioresources, IBA Bucharest, 6 Dinu Vintila Street, 021102 Bucharest, Romania; alinaculetu@gmail.com (A.C.); denisa.duta@bioresurse.ro (D.E.D.); 2 Department of Food Science and Technology, International Hellenic University, P.O. Box 141, 57400 Thessaloniki, Greece; mariapapage@ihu.gr; 3 Department of Food Science and Technology, University of the Peloponnese, 24100 Kalamata, Greece

**Keywords:** gluten-free, hydrocolloids, dough rheological properties, texture, volume, sensory, glycemic index, staling, bread, pasta

## Abstract

Hydrocolloids are important ingredients controlling the quality characteristics of the final bakery products. Hydrocolloids are frequently used in gluten-free (GF) recipes, mimicking some rheological properties of gluten, improving dough properties, delaying starch retrogradation and improving bread texture, appearance and stability. Hydrocolloids addition increases viscosity and incorporation of air into the GF dough/batter. Besides their advantages for the technological properties of the GF bread, hydrocolloids addition may impact the glycemic index (GI) of the final product, thus answering the demand of people requiring products with low GI. This review deals with the application of hydrocolloids in GF bread and pasta with a focus on their effect on dough rheology, bread hardness, specific volume, staling and GI.

## 1. Introduction

Hydrocolloids are a group of water-soluble polysaccharides with different chemical structures, high molecular weight and hydrophilic long-chain molecules. Hydrocolloids’ addition has a positive impact on gluten-free (GF) cereal-based products because they improve the structure, volume, texture, taste and overall quality of the final products as well as a shelf-life extension [1,2,3].

The use of hydrocolloids in GF applications depends on their colloidal properties, the ability to increase the water-binding capacity, viscosity, hydration rate and the effect of temperature on hydration because, for most hydrocolloids, the viscosity decreases with increasing temperature [1]. Hydrocolloids also improve the development and retention of gases during fermentation.

**Figure 1:**
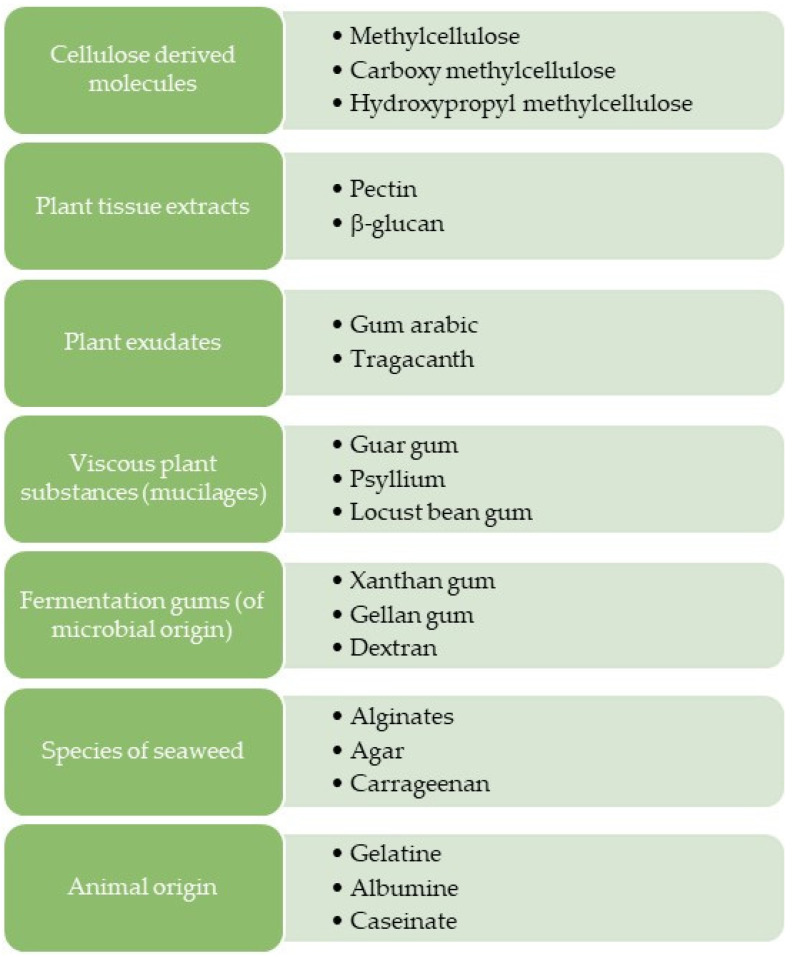
Classification of the main hydrocolloids according to their origin.

Hydrocolloids are classified according to their origin, as shown in Figure 1. Different types of hydrocolloids were used in GF products, including hydroxypropyl methylcellulose (HPMC), xanthan gum (XG), guar gum (GG), locust bean gum, psyllium, carrageenan, pectin, carboxymethyl cellulose (CMC), konjac gum, gelatine, agarose, agar, β-glucan, gum arabic (GA) and alginate [4,5,6].

Furthermore, hydrocolloids addition represents the easiest way to increase the dietary fiber content of GF bakery products. In general, GF products are characterized by a much lower nutritional value due to the fact that they lack important nutrients, such as vitamins, proteins, minerals and dietary fiber. One of these ingredients used in the food industry, classified as dietary fiber, is β-glucan, a non-starch polysaccharide that is located in the walls of endosperm cells of oats and barley. Moreover, psyllium, a natural bioactive soluble fiber that can be used as hydrocolloid replacer due to its water-holding, gel-forming and structure building properties, received attention in GF preparations in the last years. Psyllium is able to control crumb texture, as it is interchangeable with other commonly used hydrocolloids (XG, GG, HPMC) [7].

In the present manuscript, the impact of hydrocolloids addition into the formulation of GF bread and pasta products, with a focus on the dough rheology, hardness, specific volume, staling, glycemic index and sensory characteristics, are reviewed. 

**Figure 2:**
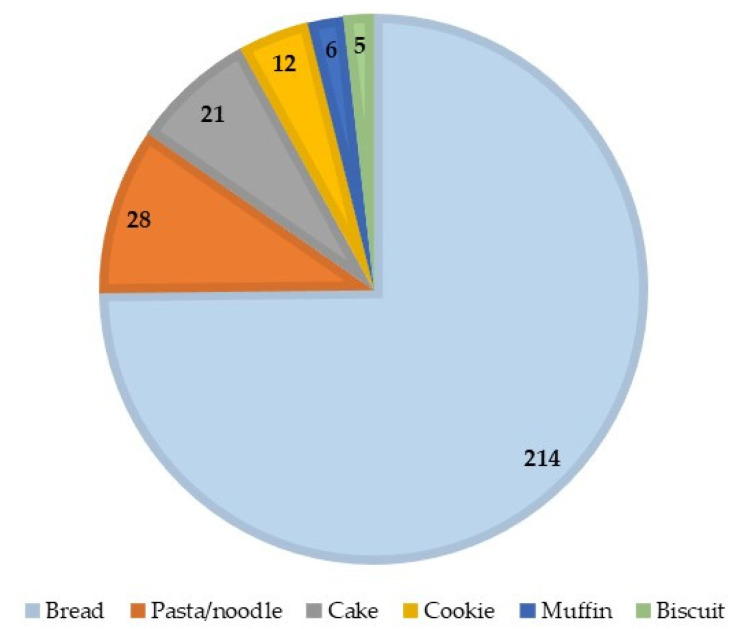
Number of publications dealing with hydrocolloid applications in different GF products. Results were obtained on 19 October 2021 on the Web of Science database.

**Figure 3:**
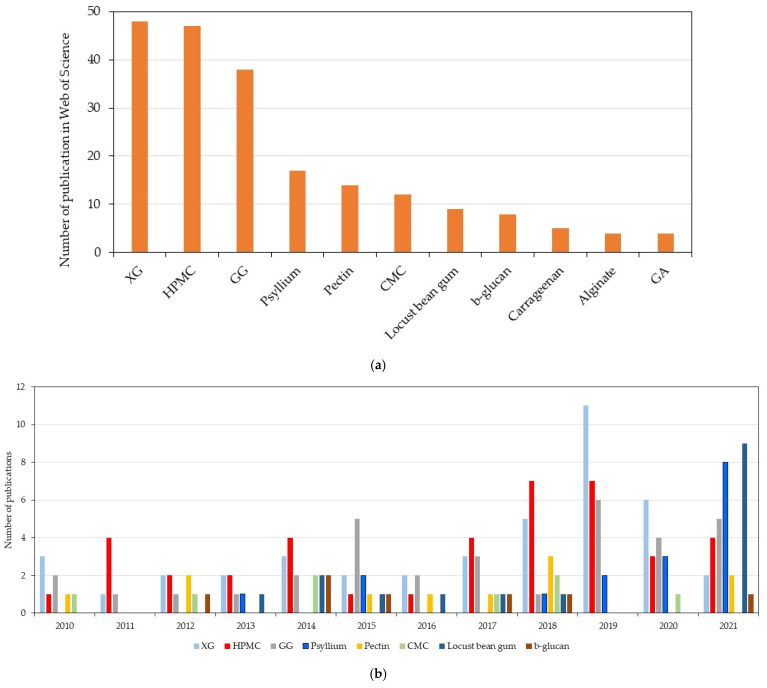
(**a**) Number of publications by name of the hydrocolloid in GF bread applications. (**b**) Number of publications by name of the hydrocolloid in GF bread applications over time (in the last 11 years). Results were obtained on 19 October 2021 on the Web of Science database.

A comparison of articles from the Web of Science database by using the terms “gluten-free bread/pasta/noodles/cake/cookie/muffin/biscuit” in the article title AND “hydrocolloid” as well as the exact name of each of the following hydrocolloids in the abstract: XG, HPMC, GG, psyllium, pectin, CMC, locust bean gum, β-glucan, carrageenan, alginate, GA (document type: articles and review articles; language: English; no other exclusion criteria), showed a significantly higher number of papers published for bread as compared with the other GF products, followed by those addressing pasta products (Figure 2). This is explained by the fact that the gluten absence is critical in GF breads in regard to the bread structure, which makes it more challenging to find new approaches to improve the bread properties. Figure 3a shows the number of publications for GF bread according to the name of the hydrocolloids, while the papers’ distribution over time is shown in Figure 3b.

XG and HPMC are the most frequently employed hydrocolloids for GF breads, mainly for their impact to increase the volume and porosity as well to produce softer products, followed by GG and psyllium. A higher frequency was noted in the last 3 years for psyllium application in GF bread. Psyllium is a promising addition to improve GF bread, enhancing the volume, structure, texture, appearance and acceptability of GFB, in addition to increasing the dietary fiber content and decreasing the glycemic response of GF bread [7]. 

## 2. Hydrocolloids in GF Bread

In GF doughs, hydrocolloids are used to create a viscoelastic network in order to balance the lack of gluten. Comprehensive reviews about the impact of the hydrocolloids on dough handling, technological and nutritional properties of GF breads underlined their function as structuring agents, mimicking the gluten network because of the ability to bind water [2,4,5,8]. In addition, hydrocolloids bring positive effects on the viscoelastic properties of the GF dough and bread texture [8].

A recent review stated that HPMC is the most favorable hydrocolloid in GF bread manufacturing [9]. HPMC forms a gel network on heating and shows lower variability than other hydrocolloids [10]. The presence of HPMC in the GF system makes the starch granules adhere to one another, and there is more space to entrap water in the system [4]. HPMC, together with the components from the rice flour, form hydrophilic bonds that are beneficial to the water absorption and contribute to the stability and homogeneity of the GF dough [11]. Factors that are related to HPMC functionality were related to the type of flours used, the presence of other ingredients and the percent of methoxyl groups contained in the HPMC molecule [12]. Besides the HPMC addition and hydration levels, Morreale et al. [11] pointed out the importance of HPMC viscosity to obtain GF rice breads with optimal quality.

The charge and the molecular weight of the hydrocolloids are amongst the main factors that influence bread quality [4,13]. The polar charge has an effect on the water affinity. Negatively charged hydrocolloids are more prone to build intermolecular hydrogen bonds with water, while uncharged hydrocolloids have intramolecular hydrogen bonds that reduce the interactions with water [4]. In a GF bread formulation based on potato starch, Horstmann et al. [13] suggested that negatively charged hydrocolloids such as sodium alginate and pectin create repulsive forces with negatively charged phosphate groups of the potato starch, delaying the pasting and gelatinization of starch granules, leading to lower viscosity and therefore to higher bread volume due to the high gas cell expansion. On the other hand, hydrocolloids with a neutral charge and higher molecular weight, such as GG and locust bean gum, create hydrogen bonds with leached amylose that leads to higher viscosity, thus lowering the elasticity and decreasing bread volume due to limiting gas expansion. Moreover, the molecular weight affects the water holding capacity of hydrocolloids [4,14]. Funami et al. [14] correlated higher water holding capacity for hydrocolloids with a higher molecular weight. Because of the higher molecular weight of certain hydrocolloids (XG, CMC, agarose and β-glucan) and due to increasing concentration, Lazaridou et al. [15] attributed the reduced loaf volume in GF bread formulation based on rice flour, corn starch and sodium caseinate.

Besides the factors mentioned above, the impact of hydrocolloids on the bread quality also depends on the level of the hydrocolloid used, the type of flour and other ingredients, as well as on the interaction with other components in the GF system [2]. Regarding the presence of other ingredients, it was shown that protein addition at certain levels of addition causes antagonistic interaction with the hydrocolloids. For example, in a formulation with rice flour-cassava starch and 5% HPMC, the addition of soy protein isolate (1%, 2%, 3%) and egg white solids (5% and 10%) reduced dough stability by lowering the hydrocolloid functionality, modifying the available water within the dough, weakening the interactions between hydrocolloid and starch and, consequently, reducing the foam stability [16]. Besides HPMC, other hydrocolloids such as XG and methylcellulose were reported to be used together with rich protein sources in GF formulations [17].

Dough hydration in GF bread is an important feature of final product quality. The correct volume of water is significant for strengthening the three-dimensional dough structure [11]. It is generally known that the greater the hydration, the higher the increment of the bread volume; there is a maximum hydration level after which the dough collapses during the baking process [18]. Recently, Sahin et al. [19] proved that Farinograph was a better tool in establishing the optimal amount of water in GF rice breads with different hydrocolloids as compared to the common method that uses the calculation based on the water hydration capacity of the individual ingredients: flour, starch and hydrocolloids. The authors stated that the advantage of the Farinograph method is that it takes into account the temperature changes during mixing and its effect on hydration, simulating the real process. Moreover, the Farinograph method provides data for dough stability and development time.

The following sub-sections deal with the effect of hydrocolloids on dough rheology, bread crumb hardness, bread specific volume, bread staling and glycemic index.

### 2.1. Effect of Hydrocolloids on Dough Rheology

The rheological behavior of dough is an important topic that has drawn significant attention in the research community, as rheology is linked to baking properties and bread quality. For example, it a correlation was found between the rheological properties of dough samples, and the firmness of GF bread as higher viscoelastic values of dough resulted in bread with lower hardness [20].

Hydrocolloids improve dough development and gas retention by an increase in viscosity, which will permit the production of improved GF breads [21].

Rheological investigation of the hydrocolloids effect on GF dough is achieved not only by empirical methodologies such as farinograph, alveograph, extensograph and Mixolab determinations but also with typical rheometers through creep-recovery and oscillation tests, which include strain and frequency sweeps that allow evaluating the viscoelastic dough properties [15,22,23]. The rheometer measures the deformation energy stored in the sample during a shear process, which represents the elastic component (G’—storage modulus), while the deformation energy used up and lost during shearing represents the viscous component (G”—loss modulus) of the dough. In GF bread, an equilibrium between elastic and viscous properties is needed [15]. Atypical viscoelastic behavior is achieved when G’ values are higher than G” values, which enables gas cell expansion. 

Mancebo et al. [24] stated that the creep-recovery test might estimate the bread quality characteristics better than the oscillatory test because the low deformations used in the latter do not correspond to the real processing and baking conditions. 

Table 1 presents some results published in the literature with the effect of hydrocolloids addition on the rheological dough properties and the type of rheological test used. 

The correct selection of the hydrocolloid and the amount of water in the recipe can lead to dough properties such as the wheat-containing one. In order to obtain high-quality GF bread, a high water content of up to 150% is needed [20]. Investigating different types of hydrocolloids, Sabanis and Tzia [10] found that XG required 10% more water than HPMC, GG and carrageenan in formulations based on corn starch and rice flour due to its higher water-binding capacity. Moreover, when increasing HPMC, GG and carrageenan addition levels from 1% to 2%, the water increased from 75% to 85%. In rice flour and cornstarch-based doughs prepared with different water amounts (130–150%), Lazaridou et al. [15] reported a decrease in elastic modulus as the water amount increased.

Many research on GF dough formulations underlined that dough samples present viscoelastic properties up to 0.1% strain level and the decrease in linearity was very significant beyond 1% strain level, which indicates the breakdown of the GF dough structure [15,25]. Similarly, with GF, wheat doughs showed linear viscoelasticity at strain levels lower than 0.1–0.25% [26,27], while other systems have different viscoelastic regions; for example, zein suspensions had a linear viscoelastic region below 0.003% strain level [28].

The addition of hydrocolloids to GF dough formulations showed increased elastic and viscous moduli. The elastic and viscous moduli of GF cornbread dough are increased with hydrocolloids addition, denoting a stronger dough structure formed by entrapping gas and retaining water, thus leading to higher viscosity [25]. The authors found a higher increase for HPMC than guar-based doughs. The higher increase in moduli values produced by HPMC addition compared to other hydrocolloids was explained by its capacity to form a foam that enables it to entrap gas inside the dough structure [4]. 

The oscillatory and creep tests showed that the elasticity and resistance to deformation of GF dough formulations supplemented with hydrocolloids followed the order: XG > CMC > pectin > agarose > β-glucan [15]. The higher elasticity shown by XG was attributed to its property to form a weak gel at low shear rates.

Sciarini et al. [29] used rheology at large deformation (resistance to penetration) and small deformation (frequency sweep) to study the hydrocolloids effect on GF dough prepared with rice flour, cassava starch and soy. The first method gives information about dough resistance, and XG showed the highest resistance, followed by CMC, alginate and carrageenan. The higher resistance given by XG was explained by its capacity to embrace a helix conformation in aqueous media, which changes the molecule into a rigid form. Regarding the frequency sweep tests, carrageenan was the only hydrocolloid, which showed a significant increase in both elastic and viscous dynamic moduli compared with a control dough; XG, alginate and CMC were similar to control.

Peressini et al. [30] found that XG and propylene glycol alginate (PGA) enhanced the storage modulus of a rice–buckwheat dough, with greater effect for PGA. The rheological properties and crumb quality of dough were improved through the use of PGA, which is modified alginate characterized as amphiphilic with special surface activity and emulsifying capacity [30,31]. A mixture of hydrocolloids improves both the structure and texture of the GF bread than the use of a single hydrocolloid. Zhao et al. [31] stated that co-supported hydrocolloids (HPMC–PGA) improve the overall quality of GF bread; namely, HPMC acted as a skeleton, and PGA served as a supporting matrix. The dough structure was enhanced by the rearrangement of polysaccharide polymers.

In a formulation made with a mixture of rice and buckwheat flour, HPMC or CMC showed a reducing strength and extension of the 3D network in the dough rheological behavior. HPMC addition also showed a modification of the dough thermal behavior [23].

It is known that hydrocolloids and starches that come from various botanical sources differ in functionality and properties related to granule size, composition or morphology that influence gelatinization, respectively. Thus, in GF sorghum bread formulations, the interaction between hydrocolloids (XG, HPMC and locust bean gum) and starches (potato, tapioca and rice) revealed that the best combinations in terms of bread quality were between potato starch (xanthan, tapioca starch) HPMC and rice starch (xanthan). Doughs with lower viscosities produced loaves with better crumb grain characteristics [32].

Studying the interaction between different hydrocolloids, Mancebo et al. [24] found no synergic effects between HPMC and psyllium in GF rice bread. Both hydrocolloids increased viscoelastic moduli, but only psyllium reduced the pasting temperature and compliance values, indicating higher dough strength [24]. Psyllium has very similar rheological characteristics with XG, both being responsible for weak gelling properties. Psyllium shows important hydration capacity and gel-forming properties, able to entrap CO_2_ [18].

By adding 5.5% psyllium to a formulation based on chickpea flour, an increase in consistency was shown during the initial stages of mixing at the beginning of heating related to protein network weakening as measured by the Mixolab technique [33]. A favorable dough consistency explained the increased cohesiveness and springiness of the crumb, which are desirable outcomes in the GF bread-making process.

### 2.2. Effect of Hydrocolloids on Bread Hardness

Bread crumb hardness is an important textural attribute as it is associated with the perception of consumers for freshness as well as for its relation with product shelf life. Bread crumb texture is influenced by the ingredients and recipe used. Usually, hydrocolloid addition tends to decrease bread hardness. The type of hydrocolloid, concentration and interaction are the factors that contribute to the hardness of the bread crumb [13]. As shown in Table 2, different hydrocolloids decreased the hardness of GF bread. 

Rice bread prepared with different types of hydrocolloids showed a softer crumb than control samples without addition, and the hardness increases with the following order: mix XG–GG < HPMC < guar < XG ≈ mix locust bean gum-XG < pectin < locust bean gum. The combination of hydrocolloids with an emulsifier such as DATEM further lowered the hardness values and improved bread quality regarding the specific volume and sensory properties [20]. 

However, Calle et al. [35] showed the highest value for hardness in the case of breads prepared with HPMC, XG and GG, but they attributed this increase to the type of flour used, a rhizome flour from *Colocasia* spp. On the same level of hydrocolloids addition (2.5% reported to the amount of millet flour and tapioca starch), Chakraborty et al. [36] showed that XG decreased the bread hardness as compared to other hydrocolloids, varying as follows: GG > GA > tragacanth > XG [36]. On one side, XG was shown to have a softening effect over crumb hardness [36,37], while other studies found an increase in crumb hardness [10,15]. In line with the results of Lazaridou et al. [15] for rice-based GF bread, Peressini et al. found elevation with XG level in the crumb firmness of rice–buckwheat bread [30]. 

Differences may appear from the bread manufacturing process and especially from the amount of water used. Encina-Zelada et al. [38] also showed that higher levels of XG (3.5%) at a constant water level (90%) led to an increased crumb hardness of bread formulated with 50% rice, 30% maize and 20% quinoa flours. By increasing the water content (to 110%), the hardness and consistency were decreased, producing bread with higher specific volume and softer crumbs; however, the high amount of water yielded stickier and less viscous doughs.

The capacity of the hydrocolloids to bind water helps to avoid water loss during bread storage. Sabanis and Tzia [10] found that the crumb hardness increases in the following order: HPMC < GG < carrageenan.

At a higher concentration of GG, the hardness of GF cheese bread decreased. A mixture of GG and HPMC led to an increase in bread hardness, which was explained by the water competition among the hydrocolloids and between the hydrocolloids and tapioca starch, the main GF ingredient [39]. 

In rice–buckwheat GF bread, the addition of XG or PGA improved crumb hardness by increasing the amount of water in the dough and, accordingly, the moisture content of the crumb because water has a plasticizing effect on the texture properties of the crumb cell walls [30]. Propylene glycol alginate breads showed greater improvement in terms of increased specific volume, decreased crumb firmness and crumb structure than XG breads. The positive effects of PGA were explained by a combined effect of low dough viscosity and elasticity produced by the polymer and the capacity to form elastic films at the gas and liquid interface, thus protecting the gas cells from instability [30]. 

By investigating the interactions between HPMC, psyllium and water in rice bread, no significant changes were recorded for specific bread volume when HPMC addition increased from 2% to 4% at different hydration levels between 90 and 110%. An opposite effect was observed in the case of increasing psyllium addition level from 0 to 4% when bread volume decreased and hardness increased. This outcome was diminished at higher water addition levels [24]. 

### 2.3. Effect of Hydrocolloids on Bread Specific Volume

Depending on the type and level of hydrocolloid addition used and the type of formulation, the effect of hydrocolloids over the specific volume of GF breads is different. There is no general correlation between the hydrocolloid concentration and the bread volume. For example, GF formulations based on potato starch containing pectin, HPMC and XG, did not show any significant effect over the specific volume when higher levels of hydrocolloid were used; while, in formulations with locust bean gum, GG and sodium alginate, the volume was dependent on the hydrocolloid level employed [13]. Thus, bread with the highest volume was obtained using 1% XG [40], while an opposite effect was reported by Lazaridou et al. [15] when using 1% and 2% XG (Table 3). The negative effect of XG on bread volume was explained by the hydrogen bonds that are formed between the negatively charged carboxyl groups present in the XG forms and water and starch and at higher levels of gum addition, leading to a rigid gel formation [36]. XG at high levels of addition produces doughs with too high resistance and consistency, which cause limited gas cell expansion during proofing [15,30]. The swelling of the starch granules is different in the presence of XG, and the granules are covered by a gum layer that limits the swelling at high temperatures [30]. Mezaize et al. [42] also reported that the incorporation of 0.6% XG into GF bread based on rice and cornflour and potato starch did not change the volume as compared to control, as XG addition makes the dough system too rigid to incorporate gases. On the other hand, the addition of 1.9% GG and 2.3% HPMC, respectively, increased the specific volume as compared to 0.6% XG.

Another example was in the case of rice–buckwheat bread, where a level of addition of 0.5% XG gave the maximum bread volume, and a further increase in the gum concentration led to lower volume [30]. There should be a balance between the water level and the hydrocolloid concentration. Thus, to obtain higher bread volume, Peressini et al. [30] increased water level and decreased XG level. In GF formulations based on maize starch, 2% XG and 2% psyllium produced breads with similar specific volume but higher when compared to breads with 2% HPMC [18].

Sciarini et al. [40] stated that in formulations with high water content, batter consistency is strongly associated with bread volume. In their study, Lazaridou et al. [15] also reported that 1% addition of CMC, agarose and β-glucan in GF formulation significantly increased the loaf volume.

In GF cheese breads based on tapioca starch and pre-cooked corn flour, GG increased the specific loaf volume, while the mixture of GG and HPMC did not produce higher loaf volume [39].

Another study showed that HPMC was much more effective than GG in a corn-based GF bread formulation [25]. Mainly, the volume of HPMC breads was almost 1.2–1.6 times bigger than that of the control, and the increment is higher than that obtained for GG. Moreover, the addition of HPMC improved the quality of breads, which were characterized by a crumb structure more aerated, elastic and fine [25]. Breads with higher specific volume were found using HPMC and maize starch than other formulations with rice flour, which was explained by the presence of proteins that leads to a higher consistency than in the case of rice flours [18,24]. The specific volume of bread prepared with rice and corn flours and potato starch increased at 2.3% HPMC and 1.9% GG addition, respectively [42].

With the aim to investigate the most commonly used GF flours in bread manufacturing, Hager and Arendt [12] found that the volume of teff and maize breads was positively influenced by HPMC addition, the volume of rice bread decreased, and for the buckwheat bread, no effect was recorded. XG decreased the bread volume for all types of flour used. On the other hand, HPMC reduced the hardness of all the breads, while XG had a diverse role: decreasing for maize bread, increasing for teff and buckwheat breads and no effect for rice bread.

### 2.4. Staling of GF Bread in the Presence of Hydrocolloids

The fast-staling process in GF bread is an important issue. Crumb textural parameters—hardness/firmness and resilience—are used to measure crumb staling. To predict the bread shelf-life, kinetic models (i.e., Avrami model) that describe the crumb hardness are employed [7]. 

One of the aims of the hydrocolloids addition to bakery products is to improve their shelf life by retaining the moisture content and retarding the process of staling [40]. Bread staling rate is evidence of the product’s shelf life and plays a significant role in the consumers’ acceptability. Hydrocolloids influence the starch retrogradation in bread by diminishing the loss and diffusion of water from the crumb. Starch retrogradation and bread hardness are delayed as a consequence of higher moisture content in the bread [37]. 

Staling rate was calculated, reporting the difference between crumb hardness at 24 h and at 2 h after baking [19]. The staling rate of rice bread prepared with different hydrocolloids decreased in the following order: GG > locust bean gum ≈ sodium alginate > XG [19].

Increasing the level of XG from 5 to 15 g/kg flour in a GF formulation made from corn starch, rice flour, soy flour and pre-gelatinized corn starch decreased staling during storage, while CMC-containing formulae showed no significant difference after 3 days of storage at 17–20 °C [37]. Another study confirmed that the staling rate was slower in the presence of 1% XG or 1% CMC in a formulation with rice, corn and soy flours after bread storage at room temperature [40]. Formulations with the highest water content and lower moisture loss had the minimum staling. The hydrogen bonding between hydrocolloids and starch retards starch retrogradation [10].

Guar gum may also delay bread staling as it was observed in the GF cheese bread during storage for 6 days at room temperature due to its hydrophilic character that prevents water release and polymer aggregation. The mechanism proposed was based on a possible inhibition of amylopectin retrogradation as GG preferentially binds to starch [39].

Sciarini et al. [29] observed the following trend for the staling rate (related to the crumb-hardening) of bread based on rice flour, cassava starch, full-fat active soy and hydrocolloids: control > XG > carrageenan > alginate > CMC.

Moreover, the bread staling was faster with GG than sodium caseinate at a 1.5% level of addition in GF potato flour-based bread formulations because of its excessive moisture accumulation, but both hydrocolloids were effective in reducing the rate of staling when compared to the control bread. Besides the positive effect of the hydrocolloids on bread staling, benefits over the bread staling can be brought by the use of potato flour in the bread formulation due to its higher starch content and longer amylopectin side-chains, which contribute to the retaining of moisture in the bread during storage when compared to other cereals [43].

Psyllium is an effective anti-staling agent that significantly delays bread staling due to its higher capacity to bind water, limiting the water mobility, which decreases starch hydration, gelatinization and retrogradation thus, influencing the crumb hardening kinetics [7,44]. A reduction in bread staling was reported with a 17.14% psyllium addition and 117.86% water to a formulation consisting of 75% rice flour, 25% cassava starch, 25% whole egg, 10.5% whole milk powder, 6% white cane sugar, 6% soy oil, 2% salt, 0.8% dry yeast, and 0.1% calcium propionate. The authors found 75% softer crumbs in the psyllium-enriched GF bread [44]. In wholegrain buckwheat/carob-based GF bread (90.7%/7.3%), 2% psyllium addition delayed crumb hardening during 10 days of storage [7]. The staling effect was also attributed to the types of flour used (i.e., buckwheat and carob). Other studies found that chickpea flour in combination with psyllium reduced and delayed GF bread staling after 7 days of storage [33,45]. The higher fiber content from psyllium addition contributed to a greater crumb springiness and cohesiveness that inhibited the bread from crumbling during storage [33].

### 2.5. Estimated Glycemic Index of GF Bread in the Presence of Hydrocolloids

Celiac disease is associated with a high incidence of type I diabetes, and patients must maintain a constant glycemic control while adhering to a strict GF diet [46]. The glycemic index is influenced by several factors such as starch granule, bread structure and viscoelasticity. It was previously reported that the glycemic index of GF bread is much higher compared to the traditional bread, exerting an influence over chronic diseases [47,48,49]. The strategies to reduce the glycemic response of starchy gluten-free products refers to the replacement of flours and starches with alternative raw materials (characterized by an increased content of dietary fiber, protein and resistant starch), the addition of viscous dietary fibers and application of different processing conditions such as grain germination, sourdough fermentation or hydration level [50,51,52].

The use of high amounts of pure starches and rice flour in GF products determines higher glycemic index values (i.e., above 80) [53]. In GF rice bread, de la Hera et al. [54] underlined that the more compact the structure of the bread, the lower the glycemic response. In breads with higher amounts of water (90–110%), the estimated glycemic index was higher. Other alternative GF raw materials, such as *Colocasia esculenta* (a rhizome) flour, either thermally treated or in mixtures with hydrocolloids, contribute to the reduction in the glycemic index (i.e., below 30) [35].

There are few papers investigating the effect of hydrocolloids addition on the glycemic index of GF breads (Table 4). Liu et al. [41] showed that hydrocolloids addition (HPMC, CMC, XG and apple pectin) significantly reduced the rapidly digestible starch and the estimated glycemic index of the gluten-free bread based on potato flour compared to control bread. The hydrocolloid forms a layer around the starch granules, retarding the enzymatic hydrolysis and thus acting as a barrier to the enzyme attack or to the release of the products of hydrolysis [41,55]. Hydrocolloids addition modifies the starch gelatinization properties, influencing the starch digestibility. Higher percentages of hydrocolloid addition contribute to viscosity changes that cover the starch surface, preventing the α-amylase access [41]. The authors explained these phenomena for HPMC, CMC or apple pectin additions, while XG showed an opposite effect attributed to its higher molecular weight. Higher molecular weight was reported to enhance the viscosity of the liquid in the upper digestive tract, reducing the in vitro starch digestion and the glycemic response [56]. It was also reported that the addition of certain hydrocolloids (sodium carboxymethyl cellulose and XG) decreased the glycemic index of wheat-based bread [57].

Under simulated gastric and intestinal conditions, it was shown that the addition of guar gum in waxy maize starch reduced the glycemic response parameters, namely, by almost 25% in the starch hydrolysis and by 15% at the end of in vitro intestinal digestion [58]. The decreasing effect of gums over the post-prandial glycemia after ingestion of starchy foods was attributed to the gum’s capacity to induce high viscosity in the gut lumen [59]. The authors found that the consumption by a non-diabetic group of subjects of wholemeal bread made with 15% guar addition produced a significantly lower blood glucose level at 30 min compared to control bread. In addition, the plasma insulin responses at 30 and 60 min were lower in the case of 10 and 15% guar additions compared to the control.

Recently, Montemurro et al. [60] formulated a “clean-label” gluten-free bread using natural hydrocolloids (a mixture of psyllium, flaxseed and chia flours as structuring agents), rice and maize flour fortified with quinoa flour and chestnut dough containing exopolysaccharides, which showed similar in vitro glycemic index (a value of 85 calculated with wheat bread as reference) as compared to other commercial GF breads. A lower estimated glycemic index (55.2) was obtained for a GF potato steam bread, containing 4.84% pre-gelatinized potato flour, 1.68% HPMC, 5.87% egg white protein, and 69.69% water based on potato flour [61]. The value was much lower compared to the value of 73.6 for the wheat steamed bread [61].

Because different compounds (among them, fat, protein, dietary fiber, hydrocolloids, starch type) may interfere in the glycemic analysis, it is relatively difficult to compare the glycemic values between breads. Moreover, the method used plays an important role in the calculation of the glycemic index. 

Some researches focused on evaluating the influence of psyllium on the post-prandial glycemic response of GF bread [62,63]. The addition of 17.14% psyllium to a GF bread formulation based on rice flour and cassava starch exhibited a decrease in the glycemic index by 25% compared to a control bread without psyllium addition [62]. Similarly, the combination of chickpea and 5.5% psyllium in gluten-free bread-making reduced the glycemic index by 25% [63].

Besides the reduction in the glycemic response, psyllium addition enhanced the bread volume, appearance and sensory acceptability score, yielding softer crumbs as well as higher dietary fiber content.

## 3. Hydrocolloids in GF Pasta/Noodles

Pasta/noodles represent one of the most consumed GF products due to their versatility to be produced in different shapes, from various ingredients: legumes, pseudocereals, etc. Hydrocolloids play a crucial role in obtaining fresh and cooked pasta. The dough rheology during mixing, heating and cooling is influenced by the hydration during pasta preparation. The addition of hydrocolloids may affect pasta color, hardness and firmness. Table 5 presents the effect of hydrocolloid addition on some GF pasta. 

Sensory attributes of GF pasta are influenced by the nature of the raw ingredients used and the addition of hydrocolloids. GF tiger nut noodles made with XG and an adapted amount of water showed the best quality, considering the lowest cooking losses obtained and higher firmness values. Colour was differently affected by hydrocolloids addition, observing a decrease in luminosity, although significant only when hydration was adapted in the presence of XG, GG or CMC [64]. The authors stated that GG, XG and CMC, increased the noodles diameter while the level of hydration influenced the rheological behavior due to the high ability to retain water.

Padalino et al. [65] evaluated the following sensory attributes of GF spaghetti: color, homogeneity, odor, overall quality for noncooked spaghetti and elasticity, firmness, bulkiness, adhesiveness, color, homogeneity, odor, taste and overall quality for cooked spaghetti. The best overall quality was obtained by the addition of 2% CMC or chitosan. Moreover, pasta based on maize and oat flours with added chitosan as hydrocolloid showed an increased content of water-insoluble fibers, which is beneficial for reducing the glycemic index; spaghetti with CMC and agar, on the other hand, returned an increased water-soluble fiber content, which makes them recommended for reducing the blood cholesterol level.

Pasta prepared with 1.0% GG and amaranth flour showed higher sensory scores for firmness, texture, taste and overall quality of pasta [67].

In GF pasta with cassava starch and cornflour, XG improved dough handling and a level of addition of 0.6% had the highest potential to improve the pasta capacity to prevent structure disintegration, showing the lowest cooking loss and the lowest values for firmness, cohesiveness, chewiness, springiness and cutting force as well as a non-adhesive mouthfeel [69].

De Arcangelis et al. [70] prepared innovative GF pasta with the highest cooking quality and texture using a combination of 0.1% PGA, 0.5% monoglycerides of fatty acids and the gelatinization of a mixture of flours (buckwheat, maize and rice).

## 4. Conclusions

Hydrocolloids are widely used in GF systems to increase: dough handling properties, viscosity and incorporation of air into the GF dough/batter, overall quality and to extend the shelf-life of final products as a result of their structure-building and water-binding properties. Most of the hydrocolloids benefits are explained by their property to increase the water-holding ability of the dough system due to high molecular weight that helps to create a more stable structure.

In GF bread, hydrocolloids are used as gluten replacements and stabilizing agents. Furthermore, hydrocolloids can delay the release of digested carbohydrates and, thus, decrease the glycemic bread index. Among the hydrocolloids that reduced in vitro starch digestibility and estimated glycemic index are: HPMC, CMC, XG, apple pectin or psyllium, depending on the addition level in the GF formulations.

The positive effect that the hydrocolloids addition brings to the GF dough matrix depends not only on the type and concentration used but also on the interactions with the flour and other ingredients as well as on the process parameters (temperature, pH). XG and HPMC are the most employed hydrocolloids for GF breads. In GF pasta, hydrocolloid addition is used to improve dough handling, cooking quality and texture, as well as to obtain higher sensory scores. There is a lower number of publications that study the impact of hydrocolloids on the batter rheology of GF sweet products as compared to GF bread.

## Figures and Tables

**Table 1:** Effect of hydrocolloids on dough rheology.

Type of Hydrocolloid	Level Used *	Other Ingredients	Type of the Rheological Test	Effect	References
GG	1%	chestnut flour with 4% chia flour	Creep-recovery (rheometer)	Improved the dough elasticity by 65.9%	[34]
HPMC	2%			Improved the dough elasticity by 64.8%	
Tragacanth gum	1%			Improved dough elasticity by 45.8%	
XG–GG (mix)	0.5%	100% rice flour, 8% sugar, 8% shortening, 2% salt, 1% instant yeast, 150% water	Frequency sweep	Increased elastic and viscous moduli	[20]
CMC	1%	70% rice flour, 30% buckwheat flour, 85% water	Frequency sweep	Increased complex modulus, improved the internal structure, increased the crumb porosity, similar to the standard wheat bread	[23]
HPMC	1%	70% rice flour, 30% buckwheat flour, 85% water			
HPMC	1%	70% rice flour, 30% buckwheat flour, 100% water			
HPMC	1–1.5%	75% corn starch, 25% rice flour, 2% yeast, 4% sunflower oil, 4% sucrose, 2% salt, 75–85% water	Shear properties, Power law	Improved viscosity	[10]
GG					
Carrageenan					
XG					
HPMC	5.5%	22.2% corn meal, 77.8% corn starch, 5.5% sugar, 2.2% salt, 1.1% yeast, 83.3% water	Strain and frequency sweep measurements	Increased elastic and viscous moduli	[25]
XG	4%	90% sorghum flour, 10% potato starch, 100% water, 6% sugar, 3% baking powder, 1.5% salt	RVA	Lowered viscosity 2.8 vs. 3.4 cP (control)	[32]
HPMC	3%	90% sorghum flour, 10% tapioca starch, 100% water, 6% sugar, 3% baking powder, 1.5% salt		3.3 vs. 3.4 cP (control)	
XG	3%	90% sorghum flour, 10% rice starch, 100% water, 6% sugar, 3% baking powder, 1.5% salt		3.0 vs. 3.4 cP (control)	
Psyllium and HPMC	0–4% and 2–4%	100% rice flour, 3% yeast, 1.8% salt, 10% oil, 5% sugar, 90–110% water	Dynamic oscillatory and creep-recovery test	Psyllium incorporation reduced the pasting temperature and compliance values and increased elastic and viscous moduli	[24]
XG	0.5–1.5%	60% rice flour, 40% buckwheat flour, 1.5% salt, 4.4% oil, 5.3% yeast, 80–90% water	Frequency sweep test	Elastic modulus from 4 to 22 times higher than control	[30]
PGA	0.5–1.5%			Elastic modulus from 1.5 to 3 times higher than control	
XG	0.5%	45% rice flour, 45% cassava starch, 10% soy flour, 2% salt, 2% shortening, 3%yeast, 75% water	Large deformation and frequency sweep	Resistance: 35.6 vs. 46.3 g (control)	[29]
Carrageenan	0.5%			Increased moduli Elastic: 60.8 vs. 29.7 kPa (control) Viscous: 12.9 vs. 6.8 kPa (control)	
XG, CMC	1% and 2%	rice flour, corn starch, sodium caseinate, fresh yeast, sunflower oil, salt, sugar, 140–150% water	Oscillation measurements	Increased elasticity	[15]

* based on flour weight basis.

**Table 2:** Effect of hydrocolloids on bread hardness compared to control.

Type of Hydrocolloid	Level Used *	Other Ingredients *	Hardness, g or N **	References
Carrageenan	0.5%	40% rice flour, 40% corn flour, 20% soy flour, 2% salt, 2% shortening, 3% compressed yeast, 158% water (flour basis).	818 vs. 720 g	[40]
Alginate	0.5%		723 vs. 720 g	
XG	0.5%		402 vs. 720 g	
CMC	0.5%		639 vs. 720 g	
Gelatine	0.5%		730 vs. 720 g	
HPMC	2%	100% potato flour, 70% water, 1% yeast	28.9 vs. 58.3 N	[41]
CMC	1%		32.7 vs. 58.3 N	
XG	2%		24.1 vs. 58.3 N	
Apple pectin	1%		33.6 vs. 58.3 N	
HPMC	2%	100% rhizome flour, 227% water, 1.5% salt, 3% yeast, 2% sugar, 2% oil	316 vs. 263 g	[35]
HPMC, XG, GG	0.29%, 0.21%, 0.50%		323 vs. 263 g	
XG	1.5%	58.3% corn starch, 25% rice flour, 16.7% soy flour, 3.3% pre-gelatinized corn starch, 3.3% vegetable oil, 1.7% egg white, 1.6% salt, 1.6% sugar, 1.3% yeast, 0.42% sodium stearoyl lactylate	5.1 vs. 26.2%	[37]
XG, CMC	1%, 1%		5.7 vs. 26.2%	
HPMC	1.5%	75% corn starch, 25% rice flour, 2% yeast, 4% sunflower oil, 4% sucrose, 2% salt, 80% water	2.96 vs. 4.9%	[10]
GG	1.5%		3.46 vs. 4.9%	
Carrageenan	1.5%		3.94 vs. 4.9%	
GG	5%	100% fresh cheese, 50% tapioca starch, 20% pre-cooked corn flour, 10% margarine, 6% sugar, 97% milk	16.5 vs. 20.0%	[39]
XG	0.5%	45% rice flour, 45% cassava starch, 10% soy flour, 2% salt, 2% shortening, 3% yeast, 75% water	162 vs. 249 g	[29]
CMC	0.5%		113 vs. 249 g	
Carrageenan	0.5%		132 vs. 249 g	
Alginate	0.5%		141 vs. 249 g	
GG	1.9%	50% rice flour, 15% corn flour, 30.6% cornstarch, 4.4% potato starch, 1.6% salt, 5.1% yeast, 5.9% oil, 83.6% g water	2.91 vs. 6 N	[42]
HPMC	2.3%		1.86 vs. 6 N	

* based on flour weight basis. ** vs. control: no hydrocolloid addition.

**Table 3:** Effect of hydrocolloids on the bread specific volume as compared to control.

Type of Hydrocolloid	Level Used	Other Ingredients *	Specific Volume, cm^3^/g **	References
Carrageenan	0.5%	40% rice flour, 40% corn flour, 20% soy flour, 2% salt, 2% shortening, 3% compressed yeast, 158% water	2.6 vs. 2.4	[40]
Alginate	0.5%		2.5 vs. 2.4	
XG	0.5%		2.9 vs. 2.4	
CMC	0.5%		2.6 vs. 2.4	
Gelatine	0.5%		2.5 vs. 2.4	
HPMC	2%	100% potato flour, 70% water, 1% yeast	2 vs. 1.25	[41]
CMC	1%		1.75 vs. 1.25	
XG	2%		1.85 vs. 1.25	
Apple pectin	1%		1.6 vs. 1.25	
HPMC	1.5%	75% corn starch, 25% rice flour, 2% yeast, 4% sunflower oil, 4% sucrose, 2% salt, 80% water for 1.5% hydrocolloid/85% water for 2% hydrocolloid	2.9 vs. 2.68	[10]
HPMC	2%		2.85 vs. 2.68	
GG	1.5%		2.85 vs. 2.68	
GG	2.5%	100% fresh cheese, 50% tapioca starch, 20% pre-cooked corn flour, 10% margarine, 6% sugar, 68% milk	2.4 vs. 2.1	[39]
XG	0.5%	45% rice flour, 45% cassava starch, 10% soy flour, 2% salt, 2% shortening, 3%yeast, 75% water	1.86 vs. 1.98	[29]
CMC	0.5%		2.14 vs. 1.98	
Carrageenan	0.5%		2.38 vs. 1.98	
Alginate	0.5%		1.99 vs. 1.98	
GG	1.9%	50% rice flour, 15% corn flour, 30.6% cornstarch, 4.4% potato starch, 1.6% salt, 5.1% yeast, 5.9% oil, 83.6% water	2.82 vs. 2.47	[42]
HPMC	2.3%		3.33 vs. 2.47	
CMC	1%	rice flour, corn starch, sodium caseinate, fresh yeast, sunflower oil, salt, sugar, 140% water	2.67 vs. 2.19	[15]
Agarose	1%		2.62 vs. 2.19	
β-glucan	1%		2.68 vs. 2.19	
Pectin	2%	rice flour, corn starch, sodium caseinate, fresh yeast, sunflower oil, salt, sugar, 150% water	2.52 vs. 2.21	[15]

* based on flour weight basis. ** vs. control: no hydrocolloid addition.

**Table 4:** Glycemic index for GF bread containing hydrocolloids.

Type of Hydrocolloid	Level Used	Other Ingredients	GI Value	Method *	References
None	-	100% potato flour, 70% water, 1% yeast	73.3	in vitro starch digestibility glucose	[41]
Apple pectin	0.5%		65.1		
	**1%**		**64.8**		
	2%		65.1		
HPMC	0.5%		65.0		
	1%		60.5		
	**2%**		**58.9**		
CMC	**0.5%**		**66.2**		
	1%		68.4		
	2%		66.6		
XG	**0.5%**		**62.7**		
	**1%**		**62.7**		
	2%		63.3		
None	-	100% flour (50% *Colocasia* flour blended with 50% pre-treated *Colocasia* flour), 227% water, 1.5% salt, 3% compressed yeast, 2% sugar, 2% oil	23.9	in vitro starch digestibility white bread	[35]
HPMC	2%		**23.1**		
HPMC + XG + GG	0.29 + 0.21 + 0.50%		26.2		
HPMC	1.68%	100% potato flour, 4.84% pregelatinized potato flour, 5.87% egg white protein, 69.69% water	55.2	in vitro starch digestibility	[61]
None	-	75% rice flour, 25% cassava starch, 25% whole egg, 10.5% whole milk powder, 6% white cane sugar, 6% soy oil, 2% salt, 0.8% dry yeast, 117.86% water	66.5	in vivo white wheat bread	[62]
Psyllium	17.14%		**50**		
XG + CMC	0.3%, 0.3%	75% chickpea flour, 25% cassava starch, 6% white cane sugar, 2% salt, 0.8% dry yeast, 0.1% calcium propionate, 25% whole eggs, 6% soybean oil, 125% water	79.2	in vivo rice bread	[63]
Psyllium	5.5%		**74.6**		

* Refers to the method used to determine the glycemic index and the type of the standard food used for comparison. Bold represents the lowest GI in the corresponding study.

**Table 5:** Effect of hydrocolloids in GF pasta.

Type of Hydrocolloid/Obtained Product	Level Used	Other Ingredients	Type of the Rheological Test	Effect	References
XG, GG, CMC/noodles	0.5%	Tiger nut flour	Mixolab rheological behavior: mixing, heating and cooling consistency, extrusion force	Improved dough extensibility; XGgavehigher firmness, reduced adhesiveness, increased chewiness and resilience	[64]
Gellan Gum, CMC, Pectin PEC, Agar, Tapioca starch, Guar seed flour and Chitosan/spaghetti	2.0%	Maize flour and naked oat	Elongation and shear viscosity (capillary rheometer)	Improved cooking quality and texture properties (adhesiveness, cooking loss, hardness). Chitosan: reduced glycemic index. CMC and agar: reducing the blood cholesterol.	[65]
XG/noodle	5%	Rice flour, glutinous rice flour	Pasting properties (RVA); Frequency sweep test (controlled-stress rheometer); Dough development characteristics: water absorption, development time, stability, softening (DoughLab equipment)	Enhanced tensile strength, peak viscosity, gel strength. Increased chewiness and hardness.	[66]
GG, gum acacia and gum tragacanth/pasta	0.5–1%	Amaranth flour	Pasting properties (RVA)	GG and gum tragacanth: increased peak, trough, breakdown and final viscosities. Gum acacia: reverse trend.	[67]
GG, XG, sodium alginate	1% and 2%	Proso millet flour	Frequency sweep tests (controlled stress rheometer)	Improved dough rheology (increased viscosity and elasticity at 2% addition) Improved pasta network strength by GG and XG addition	[68]

## Data Availability

Data are contained within the article.

## Abbreviations

CMC	carboxymethyl cellulose
GA	gum arabic
GF	gluten-free
GG	guar gum
GI	Glycemic Index
G’	elastic (storage) modulus
G”	viscous (loss) modulus
HPMC	hydroxypropyl methylcellulose
PGA	propylene glycol alginate
RVA	Rapid Visco Analyzer
XG	xanthan gum
